# Discrete derivative: a data slicing algorithm for exploration of sharing biological networks between rheumatoid arthritis and coronary heart disease

**DOI:** 10.1186/1756-0381-4-18

**Published:** 2011-06-23

**Authors:** Guang Zheng, Miao Jiang, Xiaojuan He, Jing Zhao, Hongtao Guo, Gao Chen, Qinglin Zha, Aiping Lu

**Affiliations:** 1 Institute of Basic Research in Clinical Medicine, China Academy of Chinese Medical Sciences, Dongzhimen, Beijing 100700, China; 2 Information Science and Engineering School, Lanzhou University, Lanzhou 730000, China; 3 Shanghai University of Traditional Chinese Medicine, Shanghai 201203, China

## Abstract

**Background:**

One important concept in traditional Chinese medicine (TCM) is "treating different diseases with the same therapy". In TCM practice, some patients with Rheumatoid Arthritis (RA) and some other patients with Coronary Heart Disease (CHD) can be treated with similar therapies. This suggests that there might be something commonly existed between RA and CHD, for example, biological networks or biological basis. As the amount of biomedical data in leading databases (i.e., PubMed, SinoMed, etc.) is growing at an exponential rate, it might be possible to get something interesting and meaningful through the techniques developed in data mining.

**Results:**

Based on the large data sets of Western medicine literature (PubMed) and traditional Chinese medicine literature (SinoMed), by applying data slicing algorithm in text mining, we retrieved some simple and meaningful networks. The Chinese herbs used in treatment of both RA and CHD, might affect the commonly existed networks between RA and CHD. This might support the TCM concept of treating different diseases with the same therapy.

**Conclusions:**

First, the data mining results might show the positive answer that there are biological basis/networks commonly existed in both RA and CHD. Second, there are basic Chinese herbs used in the treatment of both RA and CHD. Third, these commonly existed networks might be affected by the basic Chinese herbs. Forth, discrete derivative, the data slicing algorithm is feasible in mining out useful data from literature of PubMed and SinoMed.

## Background

Traditional Chinese Medicine (TCM), is one of China's splendid cultural heritages [1,2] with various intelligent theoretical thinking. One important concept in TCM is called "Treating Different Diseases with the Same Therapy" (TDDST), which can be explained as that different diseases might be shown with similar TCM patterns based on TCM diagnostic information (such as symptoms, pulse feelings and tongue appearance). Therefore, they could be treated with similar therapies in TCM. For example, in Western medicine, Rheumatoid Arthritis (RA) and Coronary Heart Disease (CHD) are recognized as different diseases because they are different in etiology and pathology. However, in TCM pattern classification, they share similar TCM patterns during their development. RA and CHD, named as Impediment Pattern (Bi Zheng) and Palpitations/Angina Pectoris (Xin Ji/Xin Tong) respectively in TCM, could show similar TCM patterns in Qi deficiency and blood stasis based TCM pattern classification [2], thus the two diseases can be treated with similar therapy in this case (reinforcing the deficient Qi and dissolving the blood stasis), which has been practiced during the long-term TCM activities [2].

The successful clinical practise of TDDST in TCM may suggest the fact that there may be similar (or same) biological networks/basis in RA and CHD. Due to the complexity of biological networks/basis, the common features are impossible to be revealed by single experiment or research. Thus, they keep concealed hitherto. It can be supposed that some potential regularity might be discovered by integrated analysis of the global literatures on the two diseases, in both English and Chinese.

Although forming different theoretical systems, both TCM and Western Medicine are aiming at human health services. Thus the interdisciplinary research might lighten the cognition of health and diseases. Based on this, we retrieved data from both PubMed and SinoMed. In analyzing data from PubMed, we can calculate the biological networks/basis on Western Medicine commonly existed in both RA and CHD. From SinoMed, we can filter out the basic Chinese herbs used for the treatment of both RA and CHD. Therefore, we try to find out some common regularities between RA and CHD through the overall literatures in both PubMed (English literature) and SinoMed (TCM literature) by data mining technique. Progress in digital data acquisition and storage technology has resulted in the growth of huge databases. Thus, it is impossible for anyone to read them line by line, or record by record. Based on this, we turn to the technique of data mining. Data mining is the analysis of (often large) observational data sets to find unsuspected relationships and to summarize the data in novel ways that are both understandable and meaningful/useful to the data owner and users [3-12]. During the process of data mining, we explored databases of PubMed for English literature and SinoMed for TCM literature in Chinese.

## Results and Discussion

After data mining, we got some well-structured networks, i.e., wheel-shaped, wheel-wheel shaped, and wheels-connected shaped, etc. What's more, these networks are not only in good shapes, they might also demonstrate something meaningful in researches.

### Wheel-shaped Networks

**Figure 1:**
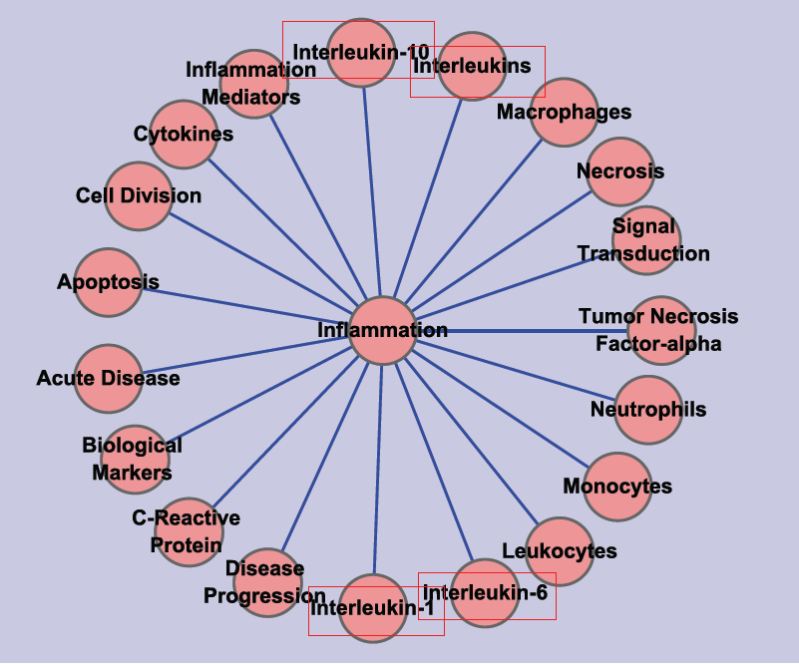
**Wheel Shaped Network on Frequency 19**. This network is constructed with data set downloaded from PubMed on May 9, 2010. It is built under the condition that any two nodes connected with each other by an edge are DescriptorName pairs on the co-occurrent frequency of "19".

In Figure 1. The network visualized in this graph shows those pairs of DescriptorNames/keywords associated with central item "Inflammation". It is clear that factors associated with inflammation are far more than those listed in this graph, in either RA or CHD. However, after associating together keywords of "Rheumatoid Arthritis" and "Coronary Heart Disease", on the frequency of "19", only these keywords in Figure 1 are left.

To be more precise, take "Interleukin" confined within rectangles for example. If only for RA, besides interleukin-1, 6, and 10, we can retrieve interleukin-2, 5, 7, 8, 11, 12, 13, 15, 16, 17, 18, and 23. It is similar with CHD, apart from interleukin-1, 6, and 10, there are interleukins of interleukin-2, 8, and 18 are concerned.

At the first glance, one may wonder that why interleukin-2, 8, and 18 are not covered by the intersection of CHD and RA. The reason is: for DescriptorNames in CHD_RA, things under considerations are the co-occurrent both in CHD and RA. Interleukin-2, 8, and 18 do exist in data sets of CHD and RA. However, there is no co-occurrent DescriptorName pairs link them into each other on frequency "19". What's more, under the framework of TDDST in TCM theory, networks of this kinds or alike can suggest something new for the research both on treatment and pathogenesis of CHD and RA.

That is to say, through the calculation of data mining, we can get something which might be useful for further research on the treatment and pathogenesis of CHD and RA.

**Figure 2:**
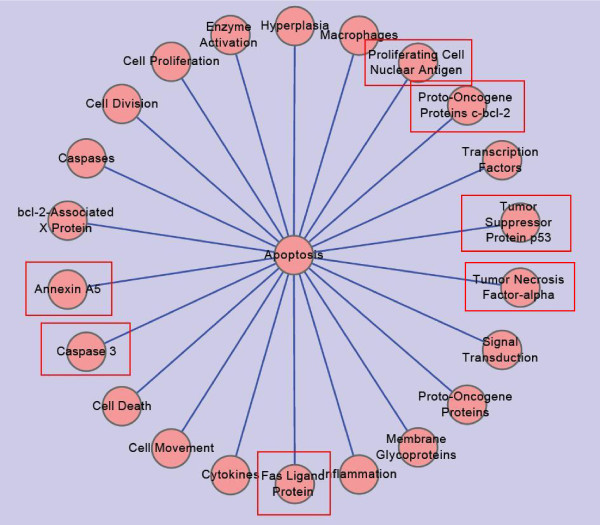
**Wheel-shaped Network on Frequency 22**. This network is constructed with data set downloaded from PubMed on May 9, 2010. It is built under the condition that any two nodes connected with each other by an edge are DescriptorName pairs on the co-occurrent frequency of "22".

Apart from "Interleukin", there are nodes tagged with "Cell Division", "Apoptosis", "Acute Disease", "Biological Markers" that are connected with center node "Inflammation". They are all important concepts in systems biology. However, tags of "Cell Division", "Biological Markers", and "Acute Disease" are not meaning too much concerned with both RA and CHD, while "Apoptosis" and "Inflammation" are more meaningful to them. Things demonstrated in Figure 1 can also be shown in Figure 2.

In Figure 2., among all the nodes connected with center node "Apoptosis", they can be grouped into two classes. One group is those nodes confined by rectangles. These nodes are more specific, usually indicating single substance which might be more meaningful in practice. The other group is those common nodes, tags of them are much more abstract. Each of them indicates a concept of a biological process, or a set of chemical material. Nodes within this group indicate knowledge in a much more abstract way, usually, they just indicate common sense.

### Wheel-wheel Shaped Networks

**Figure 3:**
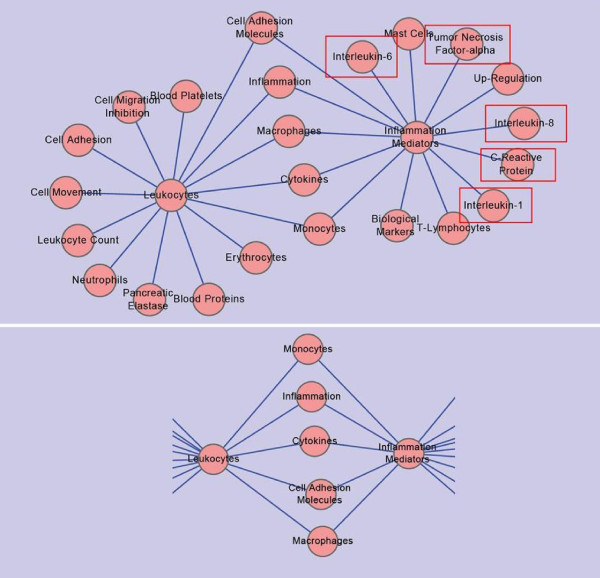
**Wheel-wheel Shaped Network on Frequency 14 and It's Sub_network**. This network is constructed with data set downloaded from PubMed on May 9, 2010. It is built under the condition that any two nodes connected with each other by an edge are DescriptorName pairs on the co-occurrent frequency of "14". The upper network in this figure is the whole network, while the lower network is a sub-network which is composed of all nodes connecting two center nodes of "Leukocytes" and "Inflammation Mediators".

Upper part in Figure 3, we can see that two wheel-shaped networks connected with each other by some commonly existed nodes.

In the left wheel, nodes around the center node tagged "Leukocytes" are all tagged with abstract concepts, i.e., "Blood Platelets", "Cell Migration Inhibition", "Cell Adhesion", "Cell Movement", and so on. As they cannot indicate much meaningful information, they are taken as noises and ignored.

In the right wheel, apart from abstract concepts (i.e., "Biological Markers", "Mast Cells", and "T-Lymphodytes"), we have "Interleukin"-1, 6, and 8, "Tumor Necrosis Factor - alpha", and "C-Reactive Protein" connected with center node with tag "Inflammation Mediators".

There is one interesting thing in this figure: an intersection composed of five nodes between two wheel-shaped networks centered by "Leukocytes" and "Inflammation Mediators". The intersection nodes are "Cell Adhesion Molecules", "Inflammation", "Macrophages", "Cytokines", and "Monocytes". What's more, the intersection nodes and the two centered nodes within wheel-shaped networks can also form a network. This network can be dug out from the inter-connected wheel-shaped network, which can is shown in lower part in Figure 3.

There are two interesting thing in this sub-network in Figure 3. One is the meaning of this sub-network which has been described above. The other is that we can get a third sub-network out from a network of two interconnected wheel-shaped networks.

Thus, we know that wheel-wheel shaped networks contain more information than single-wheel shaped network. They can also suggest much more in medical research.

### Wheels-connected Shaped Networks

**Figure 4:**
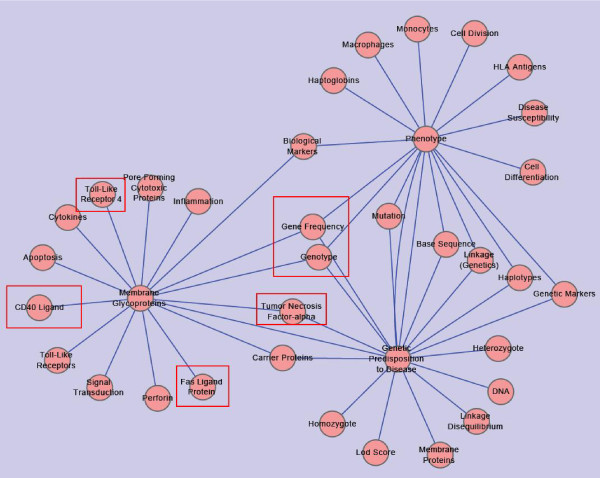
**Wheels-connected Shaped Network on Frequency 16**. This network is constructed with data set downloaded from PubMed on May 9, 2010. It is built under the condition that any two nodes connected with each other by an edge are DescriptorName pairs on the co-occurrent frequency of "16".

In Figure 4. There are three wheel-shaped networks connected with each other. Thus, they form one complex network.

This figure can tell us one important principle: all complex networks can be taken as inter-connection of single wheel-shaped networks. Of course, there might be some sub-networks which are isolated, and have no connections with others.

There are two types of inter-connections among three wheel shaped networks who are centered by "Phenotype", "Membrane Glycoproteins", and "Genetic Predisposition to Disease" respectively. One type is those nodes connects two wheel-shaped networks, i.e., "Biological Markers" connects wheels centered by "Phenotype" and "Membrane Glycoproteins", "Tumor Necrosis Factor-alpha" and "Carrier Proteins" connect wheels centered by "Membrane Glycoproteins" and "Genetic Predisposition to Disease". "Mutation", "Base Sequence", "Linkage (Genetics)", "Haplotypes", and "Genetic Markers" connect wheels centered by "Phenotype" and "Genetic Predisposition to Disease". The other type is those who connect all the three wheel-shaped networks. For example, nodes with tag "Gene Frequency" and "Genotype" confined within a red rectangle, each of them has three edges out to the three wheel-shaped networks. Apart from the two types of inter-connecting nodes, there are also meaningful nodes tagged with single chemical materials. Each of them is confined within a rectangle, i.e., "Toll-Like Receptor 4", "CD40 Ligand", "Fas Ligand Protein", and "Tumor Necrosis Factor-alpha".

### Overview of These Networks in Systems Biology

From Figure 1, 2, 3, and 4, we can find that the pathogenesis of RA and CHD are commonly associated with "inflammation", "apoptosis", "cytokines" and "macrophage" et., which also have been verified by some experimental research [13-15].

Take "inflammation" in Figure 1 for example. In the past decade, many studies reported that inflammation was the key pathogenesis in both RA and CHD [16,17]. Some immune cells and molecules such as macrophages, neutrophils, monocytes, cytokines and so on were all involved in the occurrence and development of these two diseases. The abundance and activation of macrophages in the inflamed synovial membrane/pannus significantly correlated with the severity of RA [14]. Reactive oxygen, free radicals and lipase produced by macrophges played an important role in the development of CHD [18-20]. High levels of Interleukin-1 (IL-1), Interleukin-6 (IL-6), Interleukin-10 (IL-10) and Tumor necrosis factor-*α *(TNF-*α*) in the serum were observed in RA and CHD patients [21-23]. In Figure 1, we can also find these useful information, such as "macrophage", "neutrophils", "monocytes", "IL-1", "IL-6", "IL-10" and "TNF-*α*" etc. Therefore, these data demonstrate that our method to search the commonly existed biological basis/pathogenesis between RA and CHD is feasible.

### Cross Query between SinoMed and PubMed

**Figure 5:**
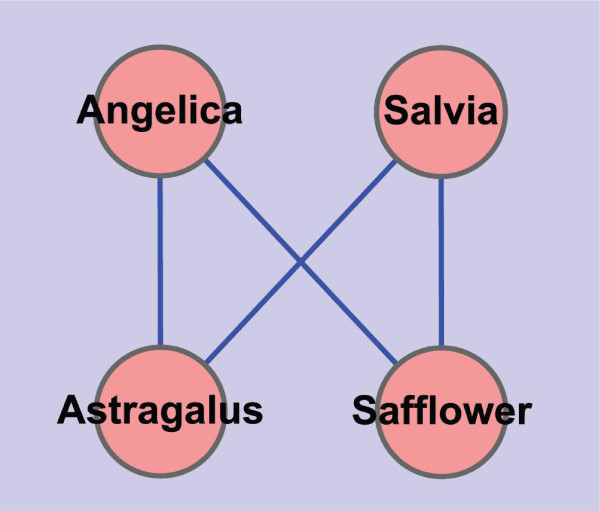
**Network of basic Chinese herbs used in the treatment of RA and CHD**. This network is constructed with data set downloaded from SinoMed on Aug. 24, 2010. First, a list of Chinese herbs pairs is built under the condition that any two nodes connected with each other by an edge are DescriptorName pairs on co-occurrent frequency. Then, supervised by TCM professionals, four basic Chinese herbs are selected out. Based on the four Chinese herbs, we mining them again in the data set of SinoMed, and construct this network based on the co-occurrent frequency greater equal than "4".

We query the keywords of "Rheumatoid Arthritis" and "Coronary heart disease" in the database of SinoMed. Several mostly used Chinese herbs for treating both of these two diseases are found. They are Angelica, Salvia, Safflower and Astragalus which can be found in Figure 5. In TCM theory, Chinese medicines of Salvia, Safflower, and Angelica can be used to treat the pattern/syndrome of blood stasis, and Astragalus can be used to treat the pattern/syndrome of Qi deficiency. What's more, in TCM practise, these four Chinese herbs can compose different couplet medicinals. By couplet medicinals, two medicinals used in pair can increase the therapeutic effect and reduce the toxic effect [24]. For example, Angelica and Salvia is more powerful in reinforcing the blood, Angelica and Astragalus can reinforce both Qi and blood, Salvia and Safflower can reinforce the blood stasis. [25].

Then, we sliced data retrieved from PubMed with Mesh terms of "Angelica", "Salvia", "Safflower" and "Astragalus" by the method of discrete derivatives and got some meaningful results shown in Table 1 and 2. Interestingly, some familiar nodes emerge again, i.e., "inflammation", "apoptosis", "cytokines", "leukocytes" and "macrophages" et al.

**Table 1:** Results of data mining on RA and CHD in PubMed and SinoMed with Chinese herbs Angelica and Salvia

SinoMed			PubMed
**Chinese Herb**	**Biology Basis**		**Detailed Information**

Angelica	Inflammation	Apoptosis	Caspase 3, Caspases, bcl-2-Associated × Protein, Proto-Oncogene Proteins c-bcl-2, Transforming Growth Factor beta1, CD40 Ligand, Tumor Necrosis Factor-alpha, Tumor Suppressor Protein p53, Vascular Endothelial Growth Factor A
		
		Leukocytes	Cyclooxygenase 1, Phospholipases A, Prostaglandin-Endoperoxide Synthases
		
		Monocytes	Interleukin-8
		
		Cytokines	Fibroblast Growth Factor 2, Intercellular Adhesion Molecule-1, Interleukin-2, Interleukin-6, NF-kappa B, Toll-Like Receptor 4, Transforming Growth Factor beta, Transforming Growth Factor beta1
		
		Macrophages	Nitric Oxide Synthase Type II, Cyclooxygenase 2, Interleukin-2, NF-kappa B, Nitric Oxide Syn-thase, Tumor Necrosis Factor-alpha, Interleukin- 6, Cyclooxygenase 1, Interleukin-1, Prostaglandin- Endoperoxide Synthases

Salvia	Inflammation	Apoptosis	Caspase 3, Caspases, Proto-Oncogene Proteins c-bcl-2, bcl-2-Associated × Protein, Tumor Suppressor Protein p53, L-Lactate Dehydrogenase, Proto-Oncogene Proteins c-akt, Proto-Oncogene Proteins, Tumor Necrosis Factor-alpha, Cyclooxygenase 2, Intercellular Adhesion Molecule-1, Interleukin-2, Interleukin-6, Interleukin-8, Platelet-Derived Growth Factor, Vascular Endothelial Growth Factor A
		
		Leukocytes	Intercellular Adhesion Molecule-1, Tumor Necrosis Factor-alpha
		
		Monocytes	Proto-Oncogene Proteins, Transforming Growth Factor beta1, Tumor Necrosis Factor-alpha
		
		Cytokines	Alanine Transaminase, Tumor Necrosis Factor- alpha, Interleukin-6, Interleukin-8, Nitric Oxide Synthase Type II, Platelet-Derived Growth Factor, Prostaglandin- Endoperoxide Synthases, Transforming Growth Factor beta1
		
		Macrophages	Nitric Oxide Synthase Type II, Tumor Necro-sis Factor-alpha, Cyclooxygenase 2, NF-kappa B, Interleukin-1, Interleukin-6, Nitric Oxide Synthase, Transforming Growth Factor beta1, Caspases, Chemokine CCL2, E-Selectin, Intercellular Adhesion Molecule-1, Matrix Metalloproteinase 9

**Table 2:** Results of data mining on RA and CHD in PubMed and SinoMed with Chinese herbs Safflower and Astragalus

SinoMed			PubMed
**Chinese Herb**	**Biology Basis**		**Detailed Information**

Safflower	Inflammation	Apoptosis	Proto-Oncogene Proteins c-bcl-2, Caspase 3, bcl-2- Associated × Protein, Caspases, Nitric Oxide Synthase, Prostaglandin-Endoperoxide Synthases, Vascular Endothelial Growth Factors
		
		Leukocytes	Interleukin-2, Leukotriene B4, Linoleic Acid, Throm-boxane B2, Triglycerides
		
		Monocytes	Interleukin-1, Tumor Necrosis Factor-alpha, Interleukin-6, NF-kappa B, Intercellular Adhesion Molecule-1
		
		Cytokines	Interleukin-6, Tumor Necrosis Factor-alpha, Interleukin-1, Interleukin-10, Thromboxane B2, 6-Ketoprostaglandin F1 alpha, Cyclooxygenase 1, Cyclooxygenase 2, Leukotriene B4, NF-kappa B, Prostaglandin-Endoperoxide Synthases, Toll-Like Receptor 4
		
		Macrophages	Eicosapentaenoic Acid, Interleukin-2, Prostaglandins E, Tumor Necrosis Factor-alpha, Interleukin-1, Leukotriene B4, Endothelial Growth Factors, Prostaglandin-Endoperoxide Synthases, Thromboxane B2, Vascular Endothelial Growth Factor A, Vascular Endothelial Growth Factors

Astragalus	Inflammation	Apoptosis	Caspase 3, Tumor Necrosis Factor-alpha, Caspases, NF-kappa B, Proto-Oncogene Proteins c-bcl-2, bcl-2-Associated × Protein, Interleukin-10, Interleukin-6, Nitric Oxide Synthase Type II, Transforming Growth Factor beta1, Tumor Suppressor Protein p53
		
		Leukocytes	Cyclooxygenase 1, E-Selectin, Intercellular Adhesion Molecule-1, NF-kappa B, Phospholipases A, Tumor Necrosis Factor-alpha
		
		Monocytes	Tumor Necrosis Factor-alpha
		
		Cytokines	Interleukin-2, Interleukin-10, Interleukin-6, Interleukin-1, Interleukin-8, Matrix Metalloproteinase 9, Toll-Like Receptor 4, Transforming Growth Factor beta1, Tumor Necrosis Factor-alpha, Vascular Endothelial Growth Factor A
		
		Macrophages	Interleukin-1, Interleukin-2, NF-kappa B, Toll-Like Receptor 4

Take "apoptosis" for example. As we know, apoptosis is the process of programmed cell death, which plays a pivotal role in tissue homoeostasis. Apoptosis disorders can lead to some serious diseases such as RA and CHD [26,27], which is also demonstrated in our study, as shown in Figure 2. We queried "Angelica", "Salvia", "Safflower" and "Astragalus" in PubMed and find "apoptosis" again and some specific nodes such as "Caspase 3", "Caspases", "Proto-Oncogene Proteins c-bcl-2", "bcl-2-Associated × Protein", "Tumor Suppressor Protein p53", et al, which are related with apoptosis and previously emerged in Figure 2. These data indicate that the mechanism of these four Chinese herbs treating RA and CHD is partly through affecting cell apoptosis by regulating apoptosis-related signal pathway, which are consistent with some experimental results [28,29].

In sum, from the Table 1 and 2, we can obtain following useful information:

1. For the treatment of RA and CHD, some basic Chinese herbal medicines are used. They are Angelica, Salvia, Safflower and Astragalus. In TCM theory, Chinese herbs of Angelica, Salvia and Safflower can be grouped into the class of reinforcing blood, while Astragalus can be grouped into the class of reinforcing Qi;

2. The mechanisms of these commonly used traditional Chinese herbal medicines for the treatment of both RA and CHD are mostly associated with "inflammation", "apoptosis", "cytokines", "monocytes", "macrophages", etc., which indicate that these herbs can affect the same biological networks that are commonly existed in RA and CHD;

3. Data mining results demonstrate that our method is feasible, and they may support the concept of TDDST in TCM;

4. These results can also give us some useful tips for the future research of these two diseases.

## Conclusions

### First, common biological networks/basis may exist

Through the calculation, we get partial results shown in Figure 1, 2, 3, and 4. These figures indicate the existence of common biological networks and biological basis in both RA and CHD;

### Second, basic Chinese herbal medicines may affect these common biological networks/basis

Through the calculation of derivatives on different orders (i.e., primary, secondary, etc.) between PubMed and SinoMed, back and forth, we get the data that support the TCM concept of TDDST through data mining. In brief, the biological basis/networks commonly existed in RA and CHD, and they might be affected by the Chinese herbal medicines which are used in TCM therapies for both RA and CHD;

### Third, our data slicing algorithm works

Through the above two items, we have reason to believe that our data slicing algorithm, together with other skills adopted in data mining, can dig out simple and meaningful information from large data sets from both PubMed and SinoMed. What's more, these results may support the concept of TDDST in TCM.

## Methods

For data mining, data preparation, pretreatment, and treatment is fundamental and necessary [30-33] for the final result.

The whole process of data mining is scheduled as described below. First, mine the data retrieved from Pubmed. Through this process, we get the common biological networks/basis existing in both RA and CHD. Then, mine the data retrieved from SinoMed. By doing this, we get the basic Chinese herbal medicines (with the highest frequencies occurring in literature) which are used for the treatment of both RA and CHD. At last, we turn to PubMed again with the basic Chinese herbal medicines for verification. After analyzing the data retrieved from PubMed with these basic Chinese herbs, we will verify our hypothesis: *these basic Chinese herbs might affect those biological networks/basis commonly existed in both RA and CHD*. In brief, we can test whether or not these Chinese herbs used against RA and CHD in TCM can affect the biological networks/basis existed in both RA and CHD.

### Data from PubMed and SinoMed

PubMed is a free database accessing the MEDLINE database of citations, abstracts and some full text articles on life sciences and biomedical topics. The United States National Library of Medicine (NLM) at the National Institutes of Health (NIH) maintains PubMed as part of the Entrez information retrieval system [34]. We retrieved data of RA and CHD from PubMed as basic material for data mining. SinoMed http://sinomed.cintcm.ac.cn/index.jsp is also a database like PubMed. The characteristic of SinoMed is that this database focus on Chinese literature in the fields of TCM, biological and medicine. We only retrieve TCM data in SinoMed for basic Chinese herbs used for the treatment of both RA and CHD.

### Query and Download Data

As mentioned above, we queried the keywords of "Rheumatoid Arthritis" and "Coronary Heart Disease" in the database of PubMed http://www.ncbi.nlm.nih.gov/pubmed/ on May 9, 2010. To be more specific, we searched MeSH for "Rheumatoid Arthritis" [MeSH Major Topic] with Restrict Search to Major Topic headings only retrieved a record set of 67,049 papers. Search MeSH for "Coronary Heart Disease" [MeSH Major Topic] with Restrict Search to Major Topic headings only retrieved records of 115,757. When this query was done, we downloaded these retrieved data sets in the type of XML into local computer system for the pretreatment.

In SinoMed http://sinomed.imicams.ac.cn/index.jsp, we queried the MeSH terms of "Rheumatoid" and "Arthritis" for RA, "Coronary Heart Disease" for CHD in Chinese on Aug. 24, 2010. By querying terms of "Default: Coronary Heart Disease or Rheumatoid or Arthritis" in Chinese, we retrieved 60,967 records for CHD, and 13,686 records for RA. The difference between PubMed and SinoMed is that PubMed can download all data in one time, while in SinoMed, we can only download 500 record one time.

### Pretreat Data

After retrieving data from PubMed (the same with SinoMed), we listed the data order by PMID and found that, for each PMID, there were several DescriptorNames associated with it. What's more, these DescriptorNames are also the keywords of this paper. Observed this, it is natural and intuitive to construct pairs of co-occurrent DescriptorNames from the retrieved data [35]. When the tables of co-occurrent DescriptorName pairs are constructed, it is natural to calculate the frequencies of these pairs [8,36,37]. When the tables of frequency pairs are built, our data slicing algorithm get its input raw material.

In order to analyze these data retrieved from PubMed and SinoMed by these given keywords, the best way is to get interesting data into the framework of structured file system, i.e., structured databases [5,7,9,38]. Because only in the structured database, large amount of optimization techniques can be applied and taking effects. Then, we can get efficient processing of our algorithm on large data sets [5].

For PubMed data sets, we transferred the XML data to the structured database of Microsoft^® ^SQL^® ^2000. In order to find new pattern and rules in data mining and text mining, we should understand our data first. The more we understand our data (especially with specified knowledge), the more accurately and efficiently we can do in further analysis [38]. For SinoMed data sets, we developed a tool to transfer its plain TXT data into Microsoft^® ^SQL^® ^2000.

In structured database, we focused on the relationships among keywords (both PubMed and SinoMed), i.e., PMID (paper ID in PubMed database) and DescriptorName (keywords associated with PMID in PubMed database). For example, we have data: *<*PMID> = '20464912', and *<*DescriptorName> = {physiopathology, rehabilitation, Evidence-Based Medicine, Humans, Muscle Stretching Exercises, Physical Fitness, Resistance Training, Treatment Outcome}. For the convenience of data processing, the set of DescriptorName is listed in Table 3.

**Table 3:** A Paper in PubMed with Its PMID and DescriptorNames

PMID	DescriptorName
20464912	physiopathology
20464912	rehabilitation
20464912	Evidence-Based Medicine
20464912	Humans
20464912	Muscle Stretching Exercises
20464912	Physical Fitness
20464912	Resistance Training
20464912	Treatment Outcome

Then, based on the assumption that the keywords' frequency can represent the research interests world wide, and potential causal connection, we construct the algorithm which is show in Table 4. This algorithm constructs the co-existing DescriptorNames in each article. Following the algorithm in Table 4 we can construct the table of co-occurrent *DescriptorName pairs *in Table 5.

**Table 4:** Algorithm of Calculating Co-occurrent DescriptorNames

USE Table I
FOR each *P MID*
*k *= Number_of_DescriptorName(*PMID*)
*j *= 1
FOR DescriptorNames(*i*) (*i *=, 1, 2,..., *k*)
DO while *j *≤ *k*
DescriptorNames Pair = DescriptorNames(*i*)+
DescriptorNames (*j*)
*j *= *j *+ 1
OUTPUT DescriptorName_Pair INTO
table DM_pairs
ENDDO
*j *= 1
ENDFOR
ENDFOR

**Table 5:** Results of Co-occurrent DescriptorName Pairs Calculated by Algorithm in Table 3 **with Raw Data Listed in **Table 2

DescriptorName_1	DescriptorName_2
physiopathology	rehabilitation
physiopathology	Evidence-Based Medicine
physiopathology	Humans
physiopathology	Muscle Stretching Exercises
physiopathology	Physical Fitness
physiopathology	Resistance Training
physiopathology	Treatment Outcome
rehabilitation	Evidence-Based Medicine
rehabilitation	Humans
rehabilitation	Muscle Stretching Exercises
rehabilitation	Physical Fitness
rehabilitation	Resistance Training
rehabilitation	Treatment Outcome
Evidence-Based Medicine	Humans
Evidence-Based Medicine	Muscle Stretching Exercises
Evidence-Based Medicine	Physical Fitness
Evidence-Based Medicine	Resistance Training
Evidence-Based Medicine	Treatment Outcome
Humans	Muscle Stretching Exercises
Humans	Physical Fitness
Humans	Resistance Training
Humans	Treatment Outcome
Muscle Stretching Exercises	Physical Fitness
Muscle Stretching Exercises	Resistance Training
Muscle Stretching Exercises	Treatment Outcome
Physical Fitness	Resistance Training
Physical Fitness	Treatment Outcome
Resistance Training	Treatment Outcome

By the algorithm in Table 4 we can build the table of co-occurrent DescriptorName pairs. By applying this algorithm with input of Table 3 we can get the result demonstrated in Table 5.

Based on Table 5 our algorithm of discrete derivatives on the distribution frequency can get its turn for calculations.

### Design Algorithm to Treat Data Sets

In the process of data mining, we construct an data slicing algorithm called *discrete derivatives*. This algorithm is based on the calculation of frequency. First, it splits data into different "layers", i.e., slicing actions are executed based on the frequency distribution of DescriptorName pairs. When data slices are calculated, we construct the function of discrete derivatives *f_dd_*. This is based on the principle of derivative in advanced calculus [39] yet in discrete space. Function *f_dd _*can calculate the difference between two adjacent data slices. When discrete derivatives *f_dd _*are calculated, we will discuss one property of function *f_dd_*.

#### PubMed Data

Among the tables of co-occurrent DescriptorName pairs in CHD, RA and CHD_RA (intersection of CHD and RA), there might be something interesting existed. In the table of CHD, there are 7,277 lines. In RA, there are 11,952 lines, and in CHD_RA, there are 1,474 lines. That is, focused on the DescriptorName pairs within both RA and CHD, there are only 1,474 pairs left. Table CHD_RA forms the initial data of un-directed graph on the DescriptorName.

Based on the table of co-occurrent DescriptorName pairs of CHD_RA in Table 5 we can further build the table of frequency distribution on it. This is done by the following algorithm in Table 6.

**Table 6:** Algorithm of Calculating Frequency of Co-occurrent DescriptorName Pairs

USE table CHD_RA
*k *= max_line_number
DO while *k *≥ 1
GO top
FOR DescriptorName_Pair(1)//The 1st pairs in CHD_RA
COUNT its Frequency
EndFor
OUTPUT DescriptorName Pairs, Frequency INTO table
CHD_RA_Frqncy
DELETE all DescriptorName_Pair(1) from table
CHD_RA
*k *= max_line_number
ENDDO

When table CHD_RA_Frqncy is constructed, we can classify the data of co-occurrent DescriptorName pairs into different slices according to their frequency distributions. Thus, we get the table of data slices as CHD_RA_Frqncy _*i *where *i *= 1, 2, 3,···, 30 with 30 is the greatest frequency existed in table CHD_RA_Frqncy.

**Figure 6:**
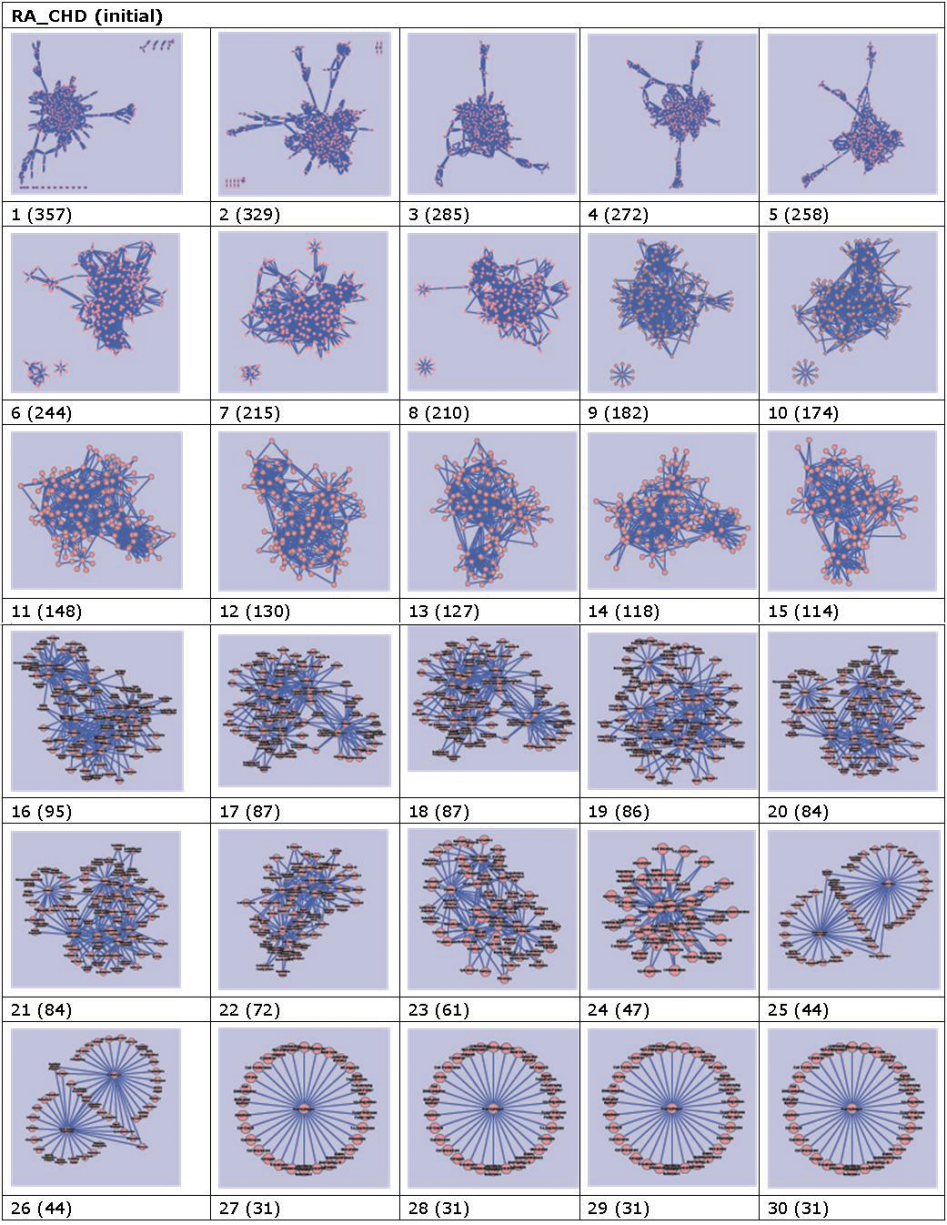
**Graphics of initial data based on frequency with nodes in parenthesis**. These networks are constructed with data set downloaded from PubMed on May 9, 2010. They are built under the conditions that any two nodes connected with each other by an edge are DescriptorName pairs on the co-occurrent frequencies. These frequencies can be demonstrated as integer which are greater equal than *i*, where *i *= 1, 2, 3,···, 30.

When this is done, we have the tables of data slices according to distributions of DescriptorName_Pairs. However, though the data slices are much more simple in complexity and small in size, we still cannot get useful data from those binary-relationship tables. Based on this, we can get them visualized for better understanding through visualization. By software Cytoscape, we can observe them in different graphs according to their frequencies, which can be shown in Figure 6.

From Figure 6, we can see that there is a trend of simplification as frequency goes from 1 to 30. For example, numbers within parentheses keep descending as frequency increases.

What's more, as frequency goes up from 1 to 18, the main part of these networks are too complicate to check for naked eye. Then, it is hard for one to get useful information on the first view. However, there are some notable "wheel" shape sub-networks of existed in graphs on frequencies of 1, 2, 6, 8, 9, and 10. These wheel-shaped sub-networks might have some meaningful cue. As frequency goes from 19 to 24, some well structured sub-networks can be observed in these graphs. For example, on frequencies of 19, 20 and 21, there are distinct wheel-shape sub-networks in the left-top position of the graphs. As frequency goes beyond 25, it is clear that we can get wonderful relationships among DescriptorNames.

Check graphs on different frequencies, we can get that, apart from the fact that most of them are distinct, there are also some similar even equivalent graphs, i.e., 25 and 26.

**Figure 7:**
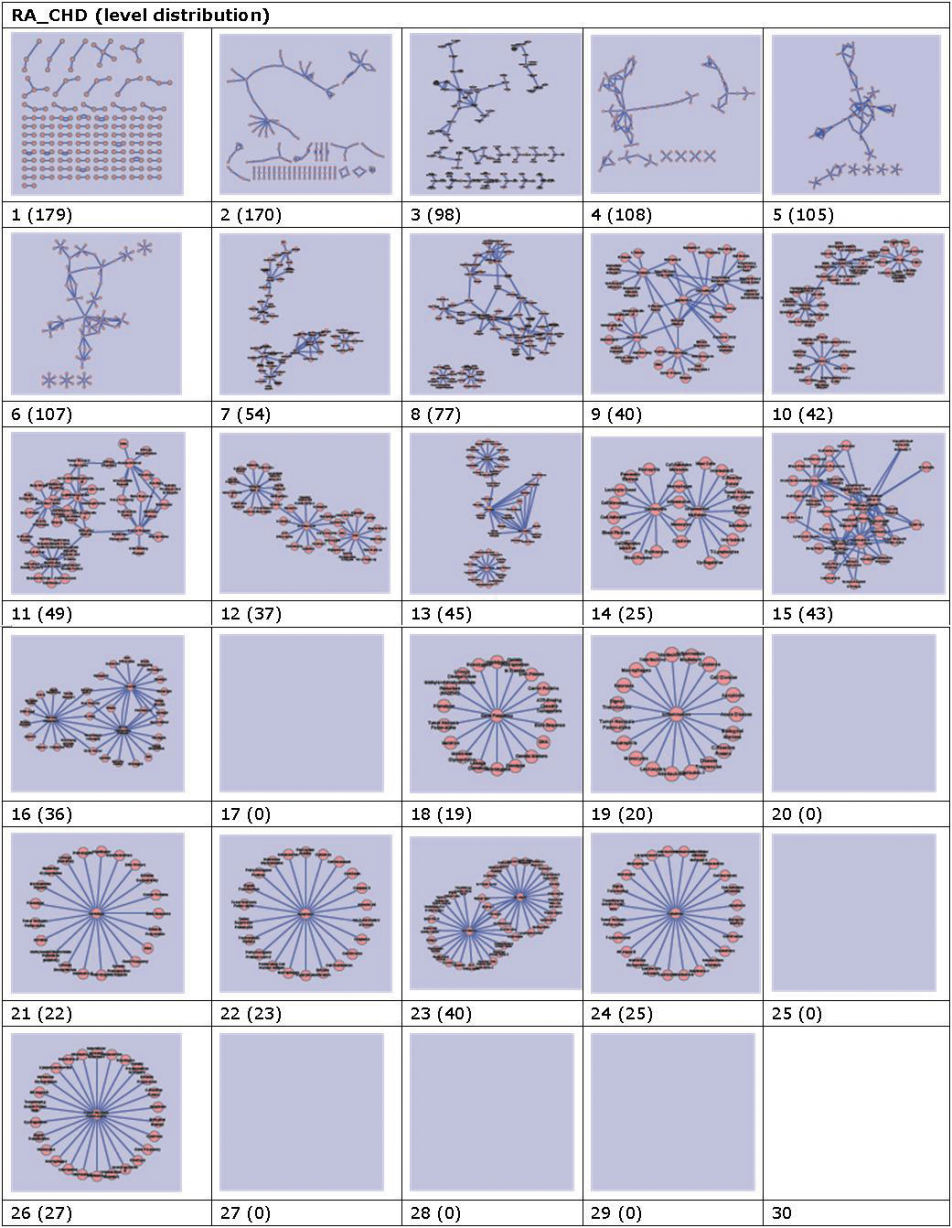
**Graphics of data slices on level distribution based on frequency with nodes in parenthesis**. These networks are constructed with data set downloaded from PubMed on May 9, 2010. They are built under the conditions that for a network on frequency *i *(*i *= 1, 2, 3,···, 30) which are in Fig. 7. We calculate the data slice *slice*_*i *_with formula *slice*_*i *_- *slice*_*i*+1 _with *i *≤ 29.

Note: in Figure 6, for better visual effects, keeping topology structure unchanged, we re-arranged the nodes in graphs on frequencies 24 and 25 by moving their positions for better view angle. For example, move nodes from density areas to sparse areas, so as to get better structured sub-networks. We also did this in Figure 7 on frequencies numbered 9, 13, 14, 16, and 23.

Now, we can calculate the distribution of co-occurrent DescriptorNames pairs on each distinct frequency. We call this distribution as *level distribution*, because it slices original data set into different subsets according to the frequency distribution, and these distributions are calculated from co-occurrent DescriptorNames pairs. Thus, level distribution is the difference between two adjacent slices of initial data set. Formally, we have the following formal definition.

**Definition 1 **(*Level Distribution*) For given data set , let *k *be the hierarchical parameter, where *k *is the maximum frequency of occurrence and *k *= *max*(*frequency*(*e*_*i*_)). For slices of data sets *slice*_*i *_(*i *= 1, 2,···, *k*) in , level distribution *ld *can be expressed by formula *ld*_*i *_= *slice*_*i *_- *slice*_*i*+1 _where *i *= 1, 2,···, *k - *1.

We calculate level distributions between different initial frequencies, i.e., top-left cell in Figure 7 with subscription of "1(179)" means the visualized data of level distribution "1". "1" means the data slice ID, and it represent the data between initial data slices of frequencies "1" and "2". "179" in parentheses means the number of nodes(DescriptorName) represented in level distribution "1". Figure 7 shows all level distributions of frequencies, i.e., from "1" to "30".

**Figure 8:**
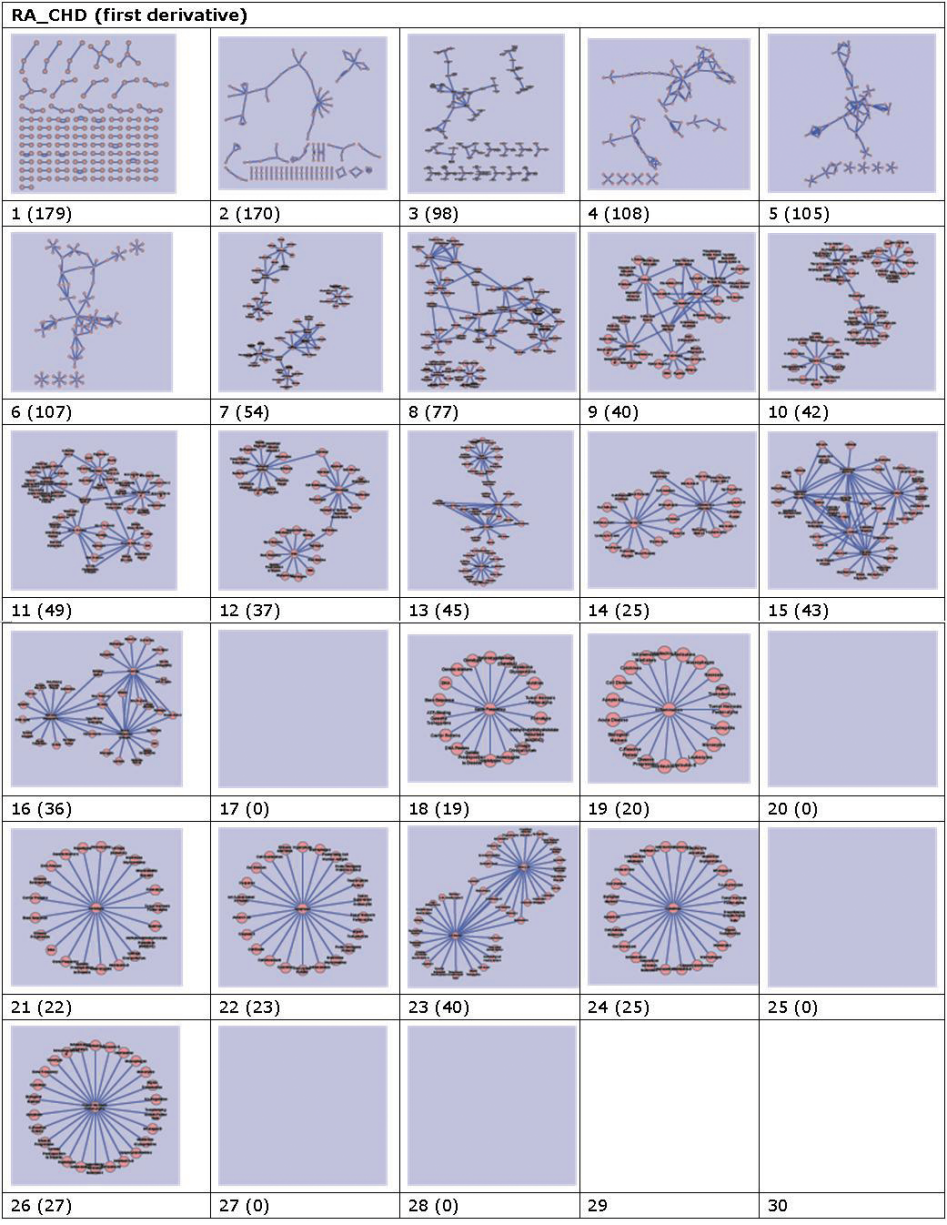
**Graphics of the first discrete derivative based on frequency with nodes in parenthesis**. These networks are constructed with data set downloaded from PubMed on May 9, 2010. They are built under the conditions that for a network on frequency *i *(*i *= 1, 2, 3,,···, 29) which are in Fig. 8. We calculate the discrete derivative  with *i *≤ 28.

From Figure 8, we can see that the level distribution of initial data is much more simple and meaningful.

Left-top graph above cell "1(179)" illuminates the data on frequency distribution of "1". There are large amount of connected pairs of co-occurrent DescriptorNames composed of "2", "3", "4" or "5" nodes. These pairs can illuminate three kinds of knowledge:

1. Common knowledge already existed, useful but meaningless for research;

2. Rarely involved knowledge, can be taken as noises or false positive findings;

3. Emerging knowledge, useful and meaningful for research.

Graphs on frequencies "1" and "2" are both of this kind. As for graphs on frequencies "3"-"8" and "15", there is no specific structures exist. To get better understanding of these data, we have to check these graphs manually. Graphs on frequencies "10"-"14", "16" and "23", are clearly structured on topology, nodes in these graphs indicate explicit relationships among DescriptorNames. As for graphs on frequencies "18", "19", "21", "22", "24", and "26", nodes inside them are wheel-shaped with a centered node, all other nodes are around the centered one. These graphs indicate some knowledge to a large probability of popular science in professional eyes. Graphs on frequencies "17", "20", "25", and "27"-"29" are blank, this means that there is no content in the level of these frequencies. On "30", as there is nothing exists higher than it but empty set ∂, so, it is meaningless to calculate its content.

As we have calculated level distribution as shown in Figure 7, it is natural for us to think of calculating the *discrete derivative *of these level distributions for even more simple results. The discrete derivative came from the principle of derivative in advanced calculus [39], which means calculating the changing-rate/velocity of the data in a continuous space. Formally, we have .

Based on this, we give out the formal definition of discrete derivative.

**Definition 2 **(*Discrete Derivative*) For a given serious of data slices *slice_i _*with *i *= 1, 2,···, *k *where *k *is a natural number, discrete derivative is .

Definition 2 gives out the definition of the first order of discrete derivative on the slices of initial data set. Enlightened by the calculation of higher order derivatives in advanced calculus [39], we give out the definitions of higher order discrete derivatives.

**Definition 3 **(*Higher Order of Discrete Derivative*)

As *ld*' = *slice*_*i *_- *slice*_*i*+1 _is the first order discrete derivative, and *ld" *= (*ld"*) is the second discrete derivative. Then, higher order of discrete derivative is *ld*^(*j*) ^= (*ld*^(*j-*1)^)*' *where *j *∈ *INT *and *j >*2.

In order to get the full view of the discrete derivatives, we calculate several orders of derivative (i.e., the first order, the second order, etc.) based on Definition 2. Most of them are similar and we show the first derivative in Figure 8.

Checking graphs in Figure 8, we can see that most of the graphs in Figure 8 are exactly the same as Figure 7, except on frequency "29". It is blanked by the definition of the algorithm on discrete derivative. It is meaningless to calculate the derivative of the last point, which is the same as frequency "30" in Figure 7. What's more, we continue our test of calculating the second, the third, and even the forth discrete derivatives, all these results demonstrate one interesting phenomenon: *for a given frequency, discrete derivatives all orders are constant*. That is to say, discrete derivatives of given frequency keep unchanged and independent of the orders of discrete derivative.

Now, take Figure 7 and 8 for example, it is very interesting that there are some slight differences between them. For example, pairs on frequencies of "2", "4", "9", "11", "12", "13",···. However, check them carefully, we can find that their nodes and edges are all the same, this means their topology of nodes and relationships are all the same.

Then, naturally, comes another question. Why graphs within these two figures under these frequencies are all the same? We all know that most derivative of functions change in the continuous space [39], i.e., , and so on. Of course, there are exceptions, i.e., *c' *= 0 where *c *is constant, (*e^x ^*)*' *= *e^x ^*and so on. These phenomena are all have their own explanations. As to the reason of our question, we believe that it is based on the essence of our data type: *discrete *characteristic data in PubMed. If we have all the DescriptorNames assigned with specific numbers in the continuous space, then, our discrete derivatives will have their numeric results. What's more, these results will change as those examples mentioned before.

Thus, we have our theorem of this interesting phenomenon.

**Theorem 4 **(*Discrete Derivatives Keep Constant*)

Given a data set  with elements *e_i _*(*i *= 1, 2,···, *n*) where  is the size of the data set.  can be made hierarchical by *k *where *k *= *max *(*frequency*(*e_i_*)), *frequency*(*e_i_*) is the occur times of *e_i _*within . Pairwise elements {*e_i_*, *e_j _*} stands for the co-occurrence within  where *i *≠ *j*. Then, discrete derivatives *ld*^(*m*) ^= *constant *where *m ≥ *1 is the order of discrete derivative.

**PROOF**: By Definition 2, we know that the first order discrete derivative is

where 1 *≤ i *≤ *k *- 1, and let .

By Definition 3, we have the second discrete derivative

By Definition 1, we have . What's more, by algorithm in Table 4 we know that *e_i _*is *distinct *co-occurrent DescriptorName pair. By algorithm in Table 6 we know that *e_i _i *= 1, 2,···, *n *are tagged with frequency *frequency*(*e_i_*). Then, *slice_i _*is the collection of co-occurrent DescriptorName pairs with *frequency*(*e_i_*) = 1.

Thus, we know that co-occurrent DescriptorName pairs of different frequency value are also distinct. That is, *ld_i _*∩ *ld_j _*= ∅ with *i *≠ *j*. Then, we have *ld_i _*∩ *ld*_*j*+1 _= ∅. Thus, we have *ld_i _*- *ld*_*i*+1 _= *ld_i _*hold, which is also the first discrete derivative . To this point, we have an important result that . Now, return to the calculation of  and , we have

and

Then, we have .

In this way, we can prove that . Together with , we have  hold, and the proof is done.

#### SinoMed Data

In order to mine out the basic Chinese herbs used in the treatment of RA and CHD in TCM, we turned to SinoMed for data sets. First, we calculated their commonly existed DescriptorNames in the same way as described in PubMed. Then, we built a table of Chinese herbs composed of 539 records as filter to filtrate the data retrieved from SinoMed. At last, we calculated the number of occurrences of Chinese herbs in the data of SinoMed. When this was done, we checked the list with TCM professionals. They found that the first four Chinese herbs listed at the top of number of occurrences are reasonable. These four Chinese herbs are "Angelica", "Salvia", "Safflower" and "Astragalus". These four Chinese herbs can be found in Figure 5. By now, we ascertain that these four Chinese herbs can be used to treat RA and CHD in TCM. However, we still do not know whether or not they have association with the knowledge hidden in PubMed which are studied by modern medical researchers. In order to verify our idea that the Chinese herbs used in both RA and CHD can affect the biological networks/basic in both RA and CHD, we explore PubMed with these four Chinese herbs.

When these data set typed XML downloaded from PubMed, we transfer them into Microsoft^® ^SQL^® ^2000 as we did before.

Through the cross queries from SinoMed to PubMed with these four Chinese herbs mentioned above, we get meaningful data in Table 1 and 2 which can support our assumption that these basic Chinese herbs used to treat RA and CHD in TCM can actually affect the biological networks/basis that exist in RA and CHD.

## Competing interests

The authors declare that they have no competing interests.

## Authors' contributions

Dr. Guang Zheng is responsible for the construction of data slicing algorithm. Dr. Miao Jiang is responsible for the explanation of traditional Chinese medicine. Dr. Xiaojuan He and Gao Chen are responsible for the explanation of western medicine. Dr. Hongtao Guo is responsible for the data collection and partial pre-cleaning from PubMed and SinoMed. Qinglin Zha is responsible for the visualization of data mining results. Prof. Aiping Lu is the supervisor of the whole study. All authors read and approved the final manuscript.
